# Methylation-related genes involved in renal carcinoma progression

**DOI:** 10.3389/fgene.2023.1225158

**Published:** 2023-08-25

**Authors:** Jose María Zamora-Fuentes, Enrique Hernández-Lemus, Jesús Espinal-Enríquez

**Affiliations:** ^1^ Computational Genomics Division, National Institute of Genomic Medicine, Mexico City, Mexico; ^2^ Centro de Ciencias de la Complejidad, Universidad Nacional Autónoma de México, Mexico City, Mexico

**Keywords:** clear cell renal carcinoma, gene co-expression networks, RAB25, ITK, IGF2BP2, TSFRN9, cancer progression stages, methylation

## Abstract

Renal carcinomas are a group of malignant tumors often originating in the cells lining the small tubes in the kidney responsible for filtering waste from the blood and urine production. Kidney tumors arise from the uncontrolled growth of cells in the kidneys and are responsible for a large share of global cancer-related morbidity and mortality. Understanding the molecular mechanisms driving renal carcinoma progression results crucial for the development of targeted therapies leading to an improvement of patient outcomes. Epigenetic mechanisms such as DNA methylation are known factors underlying the development of several cancer types. There is solid experimental evidence of relevant biological functions modulated by methylation-related genes, associated with the progression of different carcinomas. Those mechanisms can often be associated to different epigenetic marks, such as DNA methylation sites or chromatin conformation patterns. Currently, there is no definitive method to establish clear relations between genetic and epigenetic factors that influence the progression of cancer. Here, we developed a data-driven method to find methylation-related genes, so we could find relevant bonds between gene co-expression and methylation-wide-genome regulation patterns able to drive biological processes during the progression of clear cell renal carcinoma (ccRC). With this approach, we found out genes such as ITK oncogene that appear hypomethylated during all four stages of ccRC progression and are strongly involved in immune response functions. Also, we found out relevant tumor suppressor genes such as RAB25 hypermethylated, thus potentially avoiding repressed functions in the AKT signaling pathway during the evolution of ccRC. Our results have relevant implications to further understand some epigenetic–genetic-affected roles underlying the progression of renal cancer.

## Introduction

Clear cell renal carcinoma (ccRC) is the most common subtype of renal cancer. It accounts for around 75% of all cases. In spite of this, ccRC is considered a relatively low-prevalence neoplasm, with a worldwide incidence of around 2–3 cases per 100,000 people. Still, ccRC represents about 5% of all male cancer cases in the world International Agency for Research on Cancer (2023). Therapeutic options for ccRC include surgery, radiation therapy, and systemic therapy, such as immunotherapy and targeted therapy (Sternberg et al., 2010; Motzer et al., 2007; Motzer et al., 2015; Motzer et al., 2021; Kase et al., 2023).

The molecular origins of ccRC are complex and involve a number of genetic and epigenetic alterations. Perhaps, the most common genetic alteration found in ccRC is the inactivation of the VHL gene, which leads to the stabilization of a hypoxia-inducible factor (HIF) and subsequent activation of several downstream tumorigenic pathways (Varshney et al., 2017). Other molecular alterations in ccRC include mutations in PBRM1, BAP1, and SETD2, among other genes (Walton et al., 2023). Apart from these alterations, it has been known for some time that epigenomic regulation may also be playing a relevant role. Likewise, genetic abnormalities have been recognized at the chromosomal level, specifically in the short arm of chromosome 3 (in the 3p21 region) (Nabi et al., 2018). Additionally, several biomarkers for ccRC have been proposed, such as aquaporin 1 (Huang et al., 2009), perilipin 2 (Cao et al., 2018), and KIM1 (Cuadros et al., 2014), although they do not always exhibit the desired sensitivity and specificity to be clinically useful. In those terms, an accurate ccRC classification may improve the aforementioned shortcoming.

As previously mentioned, ccRC remains the most common subtype (Grammatikaki et al., 2023). For this subtype of renal carcinoma, staging of neoplasms is of great importance for prognosis, applied therapies, and the outcome of each clinical case (Moch et al., 2009). For instance, stage I has been identified as a stage with well-nourished cases and multi-omics data available in several cohorts, e.g., *The Surveillance, Epidemiology, and End Results* (SEER) and the *Fudan University Shanghai Cancer Center* (FUSCC). However, stages III and IV are marked by late diagnosis, much fewer samples, and poor prognosis. It proves the existence of subgroups in the classical classification with significantly different prognoses (Shao et al., 2018). Due to the conditions mentioned previously, it is important to understand the molecular changes occurring during transitions between ccRC stages, both as a means of prevention and to improve prognosis in the advanced stages.

Progressive features of ccRC reveal signs of its pathological complexity. Epigenomic phenomena, including DNA methylation, histone covalent modifications, and chromatin structure, as well as the regulatory activity of non-coding RNAs and their networking with each other, may play important roles in the progression of aberrant cell phenotypes. Methylation marks can control the density and compressibility of chromatin and its stability or instability for transcription, replication, and repair. A vast majority of DNA methylation (98%) occurs in CpG islands located in the promoter of certain genes in somatic cells. In cancer, associated DNA hypermethylation is influenced not only by cell-type-specific DNA methylation patterns but also by pre-existing transcriptional programs, including DNA methyltransferase malfunction (Esteller, 2002).

In terms of the regulation of gene expression, a research study has shown that the accumulation of SET oncoprotein reduces DNA methylation and histone acetylation while increasing TET1 levels (Almeida et al., 2017). However, the expression of some suppressor genes in cells with high levels of SET decreases, which suggests that methylation is not the only mechanism that regulates gene expression by this protein. This has led researchers to consider other epigenetic factors such as miRNAs and lncRNAs (Drago-García et al., 2017; Fardi et al., 2018; de Anda-Jáuregui et al., 2018; de Anda-Jáuregui et al., 2021; Zamora-Fuentes et al., 2022). Determining which cells have undergone epigenetic changes, ensuring that therapeutic agents maintain their sustainability and their capacity to penetrate the tumor mass and target malignant cells, will increase the clinical success of the treatment (Fardi et al., 2018). In concrete, identifying the epigenetic modifications driven by hypomethylation and hypermethylation in cancer may provide information relevant to the elaboration of more accurate and specific treatments.

The association between cancer-related aberrant gene expression and epigenetic modulators along with cancer progression has not been successfully unveiled. In this context, molecular data may shed light on that matter. Whole-genome gene co-expression relationships have been previously discussed, shedding light on tumor biology and even being associated with biomarkers (Andonegui-Elguera et al., 2021; García-Cortés et al., 2020; Zamora-Fuentes et al., 2020). However, the extent to which epigenomic modulation affects gene expression and co-expression patterns has been less discussed. Recent studies, however, have pointed out that epigenetic factors may be behind the progression of, for instance, ccRC (Nabi et al., 2018). In view of this, it is desirable to study the relationships between DNA methylation and gene expression patterns at a global, whole-transcriptome level in these tumors and their evolution. The results along these lines may indeed contribute to improving the prognosis and quality of life of patients in the later stages of ccRC.

Given that methylation marks are closely related to the regulation of subtle gene expression, in this work, with the main objective of providing complementary knowledge regarding the relationship between methylation and gene co-expression in ccRC progression, we constructed a fully automated method to find methylation-driven genes (MDGs) involved in ccRC progression. We developed a data-driven method to explore methylation and RNA-seq data from The Cancer Genome Atlas (TCGA) project for ccRC patients. We used the TCGA database as a source of ccRC filtered-harmonized data (Network et al., 2013). We implemented an anticorrelation model to find genes that resulted overexpressed due to significant hypomethylation. Complementarily, we observed those hypermethylated and underexpressed genes. We identified highly correlated genes to the aforementioned MDGs, and analyzed those biological processes associated to those genesets. Finally, we evaluated the key implications of these results in the context of further experimental investigation.

## Materials and methods

**FIGURE 1:**
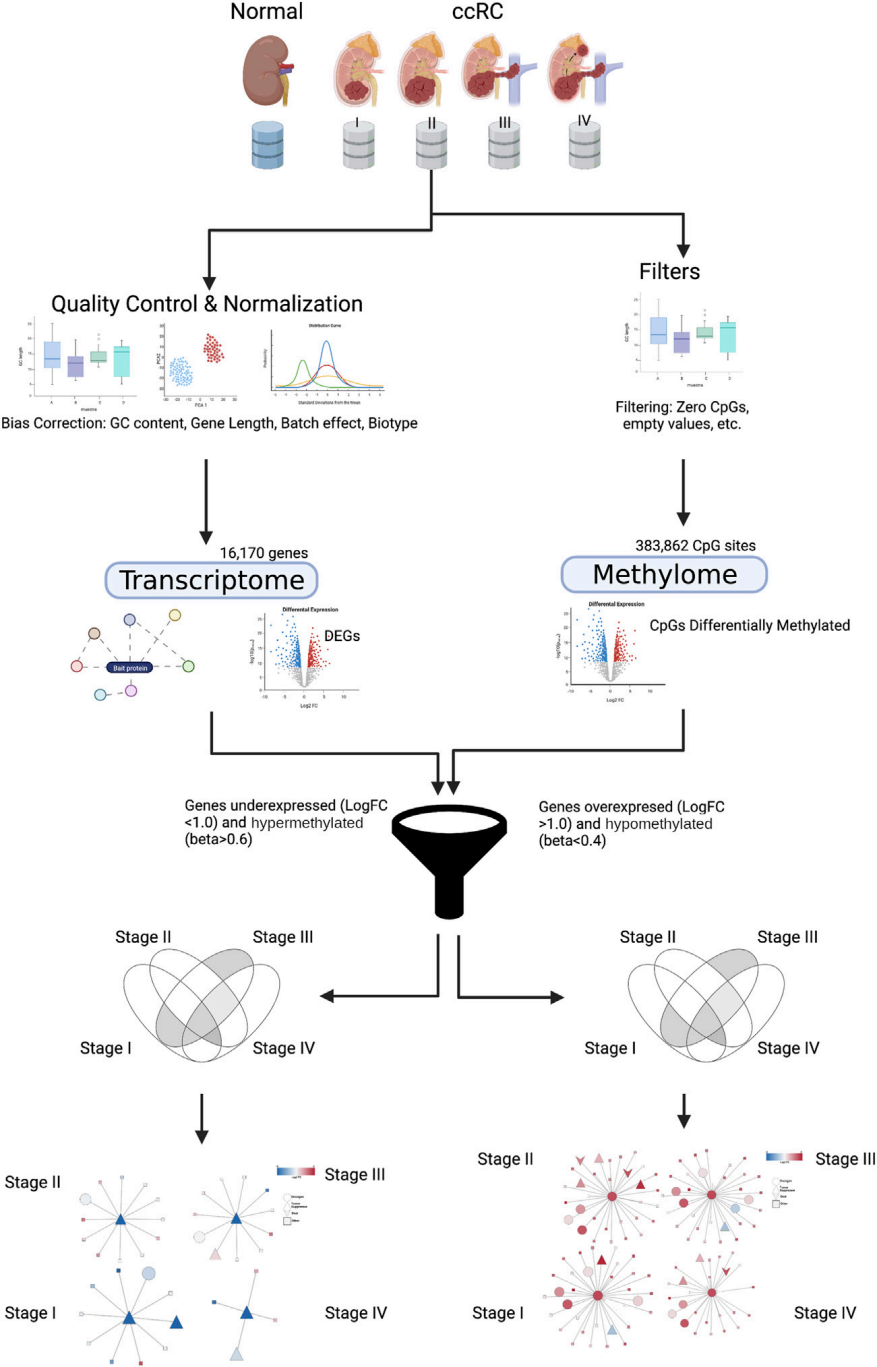
Graphical pipeline of this work. Details are explained in Materials and methods. Created with BioRender.com.

We jointly analyzed DNA methylation for 383,862 CpG sites and gene expression data for 16,170 genes coming from both ccRC and the normal adjacent tissue. We split the ccRC samples according to the progression stage: 24 non-tumor, 158 samples for stage I, 31 for stage II, 72 for stage III, and finally, 57 for stage IV. A graphical representation of this workflow is shown in Figure 1.

### Data acquisition

We used data from TCGA collaboration as a source of gene expression (from RNA sequencing) and DNA methylation (from high-density methylation arrays). In order to obtain the gene expression profiles corresponding to each progression stage, we downloaded Illumina RNA-Seq level 3 gene expression files from TCGA–ccRC samples.

We used methylation data from TCGA in the form of beta values (*β*), which measure the level of DNA methylation at known CpG sites via Illumina HumanMethylation450 (HM450) arrays. These values are calculated from array intensities (level 2 data) as $\frac{M}{M+U}$ , where *M* corresponds to methylated probes. Meanwhile, *U* takes account for non-methylated ones, marked by bisulfite conversion (Zhou et al., 2016). The indexes of both datasets were harmonized to match patient codes as a key for paste RNA-seq and methylation beta values. This is the reason for the number of samples not corresponding with the original RNA-Seq number of samples. Download, annotation, and low-level analysis were performed using the TCGAbiolinks R library (Colaprico et al., 2015). We processed the clinical information directly from the TCGA-KIRC project. We categorized all samples by *tumor_stage* variable. The samples were cleaned to exclude those samples with non-reported stages or values. The TCGAbiolinks library was also used to retrieve clinical data from TCGA.

### Data pre-processing

We pre-processed RNA-seq data as follows: 1) we removed genes without annotation in BioMart (Smedley et al., 2015), 2) we removed genes with more than 50% of zero counts per sample, and 3) genes with the mean expression less than 10 counts were removed. For sequencing bias corrections, we used the EDASeq R package (Risso et al., 2011) to remove biases in the GC content, gene length, and biotype. Finally, in order to correct for potential batch effects, we used the ARSyn method, implemented in R as a function of the NOIseq library (Nueda et al., 2012). Methylation data were cleaned up by removing those CpGs with at least one missing beta value.

Association between CpGs and genes was manually performed with the first occurrence in pre-defined annotation created by TCGAbiolinks. After all filters and bias removal procedures were applied, the total number of CpGs for assessment was 383,862; meanwhile, the total number of genes was 16,170. Those entities were used to infer different CpG–gene relations and to perform the corresponding analyses.

### Differential gene expression

Differential expression analysis was calculated with the DESeq R package (Love et al., 2014). We considered differentially expressed genes (DEGs) with the following filters: *LogFC* > 2.0 and *FDR* < 0.05. We compared the non-tumor (NT) dataset with all progression stages (*st*_*I*_, *st*_*II*_, *st*_*III*_, and *st*_*IV*_). Additionally, we contrasted consecutive ccRC stages (see Results).

### Differentially methylated CpGs

Differential methylation (DM) analysis was performed using a mean-based method implemented in the TCGAbiolinks R package. We considered differentially methylated CpGs with a mean-diff cut-off of 0.15 and a *p*-value of 0.05 (Wilcoxon test). All volcano plots for each contrast (*NT*_*S*_ *tage*1, *NT*_*S*_ *tage*2, *NT*_*S*_ *tage*3, and *NT*_*S*_ *tage*4) in ccRC progression can be calculated with a source code provided by this work. The threshold for identifying DM–CpGs was a beta-value below 0.4 or above 0.6.

### Methylation-related genes

We grouped all CpGs for each gene on the promoters’ position. We evaluated whether at least one CpG for a given gene was DM, and then, we considered this gene as a candidate to be a differentially methylated gene (DMG). Other inclusive criteria in this candidate were for the gene to be differentially expressed. We considered genes as hypermethylated when their median methylation in all their CpGs was > 0.6. Conversely, a gene with median methylation < 0.4 was considered hypomethylated.

It is worth noticing this filtering procedure for the DM CpGs of a gene is taken as a preliminary proxy. However, the sufficient conditions to determine the methylation status in a gene were given by taking the median values of all promoter CpGs within each gene.

### Oncogenes and tumor suppressors

A fundamental question regarding the role of methylation marks in ccRC progression is whether or not those marks alter cancer-related genes. Therefore, we investigated those genes with the feature to be oncogenes or tumor-suppressor genes. A comprehensive list of oncogenes was obtained of the Human Oncogene database (Liu et al., 2017). The corresponding catalog of tumor suppressors was downloaded of the TSGene database (Zhao et al., 2016). We matched genes presenting the functions of oncogene as well as tumor suppressor and these were subsequently labeled as *both* in the gene-function database (Supplementary Material S1).

### Network inference

To analyze the role of DMGs and the expression program, we constructed four DMG–DMG networks for each progression contrast, which were filtered. All networks were inferred by using the Mutual Information (MI) value as a correlation measure. The MI value was calculated over the expression values of all DMG pairs (275 × 16, 227 ≈ 4.5 × 10^6^ interactions) for each phenotype. We implemented a multi-thread co-expression calculation with the ARACNe (Margolin et al., 2006) tool. The code to infer MI-based networks can be found at https://github.com/josemaz/aracne-multicore.

In addition, to validate our results, we obtained networks using a cutoff of 1,000, 10,000, and 100,000 top edges. We have previously described considerations for setting this parameter in our work (Zamora-Fuentes et al., 2020; Dorantes-Gilardi et al., 2021). Network visualization was performed with Cytoscape 3.9.1 (Shannon et al., 2003).

### Enrichment analysis

We filtered Gene Ontology (GO) results with *p*-values < 0.01 to identify statistically enriched biological functions. We performed this analysis using both the Gprofiler2 web server and Gprofiler2 R package (Supplementary Material S2.

The complete code to develop this pipeline can be found in https://github.com/josemaz/kirc-methyl. We also added a snakemake protocol there to accomplish reproducibility and improve scientific coding practices.

## Results

### First view of methylation in stages

**FIGURE 2:**
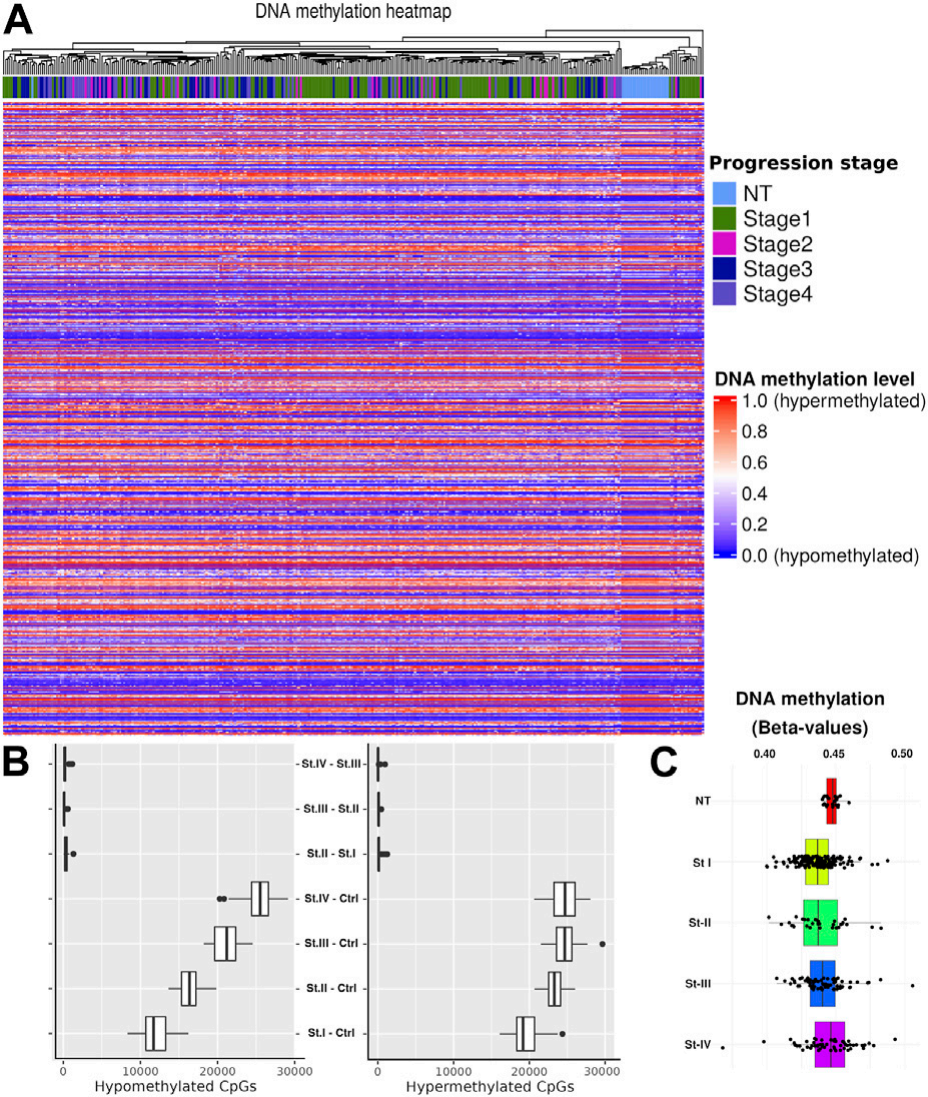
**(A)** Heatmap overview showing the methylation level for each CpG site. Hierarchical clustering was performed by the progression stage including NT. **(B)** CpG quantification by contrast. As a first approach, we considered two models of progression: 1) sequential (St1 V. NT, St2 V. St1,…) and 2) compared with control (St1 V. NT, St2 V. NT, …). It is shown that the highest amount of DM-CpGs in contrasts is given by the second model. Therefore, we adopted this second strategy. **(C)** Distributions of *β*-values in CpGs by tumor stage and for NT.

To trace changes in cancer progression associated with stages (and contrasts to normal tissue (NT)), we performed a hierarchical clustering on the data by cancer phenotype. We observed a well-defined cluster of NT samples, which is clearly separated from cancer stage samples (Figure 2A). To account for potential sample size bias, we selected subsets of methylation values of different sizes: 1*e* ^2^, 1*e* ^3^, 1*e* ^4^, and 1*e* ^5^ (Supplementary Material S3). We confirmed the same pattern, where the only samples that consistently grouped were the normal ones. Interestingly, when considering only the distributions of methylation values by the CpG site for each phenotype, it is not possible to obtain statistically significant differences between stages (Figure 2C).

Considering the previous clustering results, we calculated methylation distributions by contrast, i.e., the distribution of hypermethylated and hypomethylated CpG sites for each comparison between phenotypes. We used a bootstrap method to mitigate sample size effects (Figure 2B). In order to account for group imbalances, we used 24 samples for each phenotype in the bootstrap process, scaling down to the smallest group. The main differences were observed between the control and the different tumor stages. No statistically significant differences were observed in the progression comparisons (NT–stage1, stage1–stage2, stage2–stage3, and stage3–Stage4). Based on these results, we decided to use only the control*vs.*-each-stage contrast to develop a *progression vs. baseline model*.

### Methylation profiles are characteristic of ccRC progression stages

In Figure 2B, we can observe significant differentially methylated CpG sites (CpGs). All of these CpG sites are associated with promoter regions in several genes. This evidence led us to follow a systematic procedure to obtain genes with clear differences between phenotypes (St1 V. NT, St2 V. NT, …).

We observed a clear separation between normal tumor samples in terms of methylation. In both, the heatmap of Figure 2A and the boxplots shown in Figure 2B, the difference between consecutive progression stages is very small, contrary to the cases between the control and any stage. In addition, there is a difference between hypomethylated and hypermethylated CpGs between progression stages, increasing their number of differentially methylated CpGs according to the progression stage. This evidences methylation differences between the cancer stages. To further advance our understanding of this phenomenon, we designed a more accurate filtering method to obtain genes with methylation differences compared with the assessed contrast.

We observed a clear clustering *separatrix* between normal tumor samples in terms of methylation. In addition, there is a difference between hypomethylated and hypermethylated CpGs between progression stages, increasing their number of differentially methylated CpGs according to the progression stage. This result provides us the evidence of clear methylation differences between cancer stages. To further advance our understanding of this phenomenon, we designed a filtering method with a higher granularity to obtain genes with promoter methylation differences compared with the assessed contrast.

**FIGURE 3:**
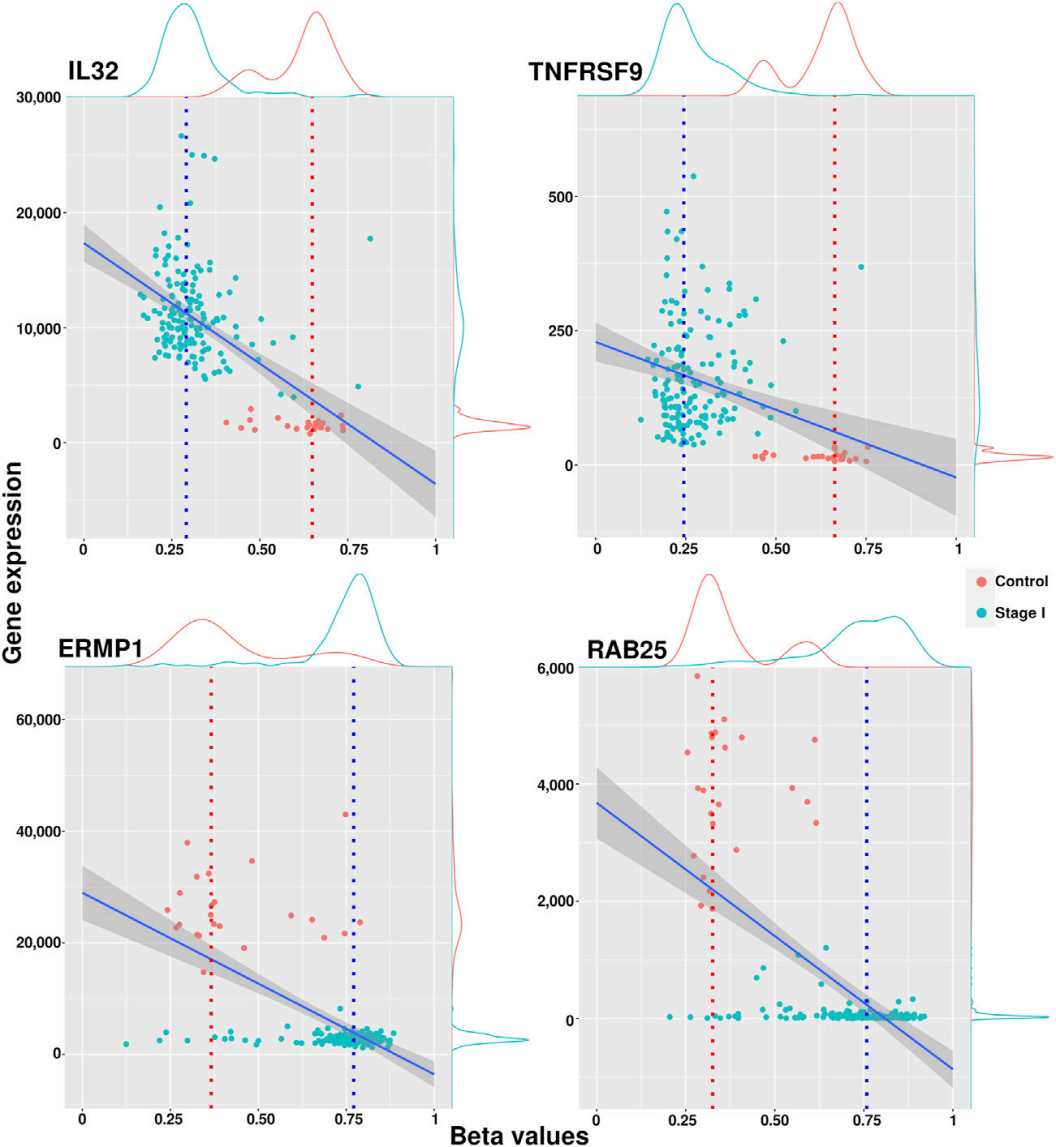
Scatter plots of methylation and expression values with examples of hypomethylated and hypermethylated genes. IL32 and TNFRSF9 have a hypomethylated condition in cancer, while ERMP1 and RAB25 resulted hypermethylated in cancer stages.

Genes with a median methylation value less than 0.4 and overexpressed were considered hypomethylated, while genes with a median methylation value greater than 0.6 and underexpressed were labeled hypermethylated. We associated these methylation modifications with phenotypic changes and assumed that they were driven by an underlying cellular mechanism that needs to be further explored. A list containing all filtered genes for each phenotype is included in Supplementary Material S4. Figure 3 shows examples of methylation behavior in two genes considered hypomethylated (IL32 and TNFRSF9) and two observed hypermethylated (ERMP1 and RAB25). The full set of scatter plots for all genes filtered by this approach can be generated with the code cited in the Data Availability Statement.

**FIGURE 4:**
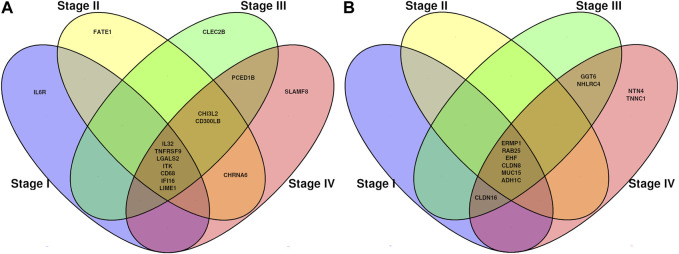
Venn diagrams showing common methylation-related genes. **(A)** Genes which decreased (hypo) their methylation pattern from normal tissue to tumor tissue. **(B)** Genes which increased (hyper) their methylation pattern from normal tissue to tumor tissue.

DMGs shared between different stages and also stage-exclusive are shown in Figure 4. We considered both the stage-specific genes for each contrast and the genes shared in all stages. Since there are no hypermethylated genes stage-exclusive in stages I, II, and III, we then analyzed those shared genes in all four stages.

### ITK is a methylation-related oncogene; RAB25 and EHF are methylation-related tumor suppressors

**FIGURE 5:**
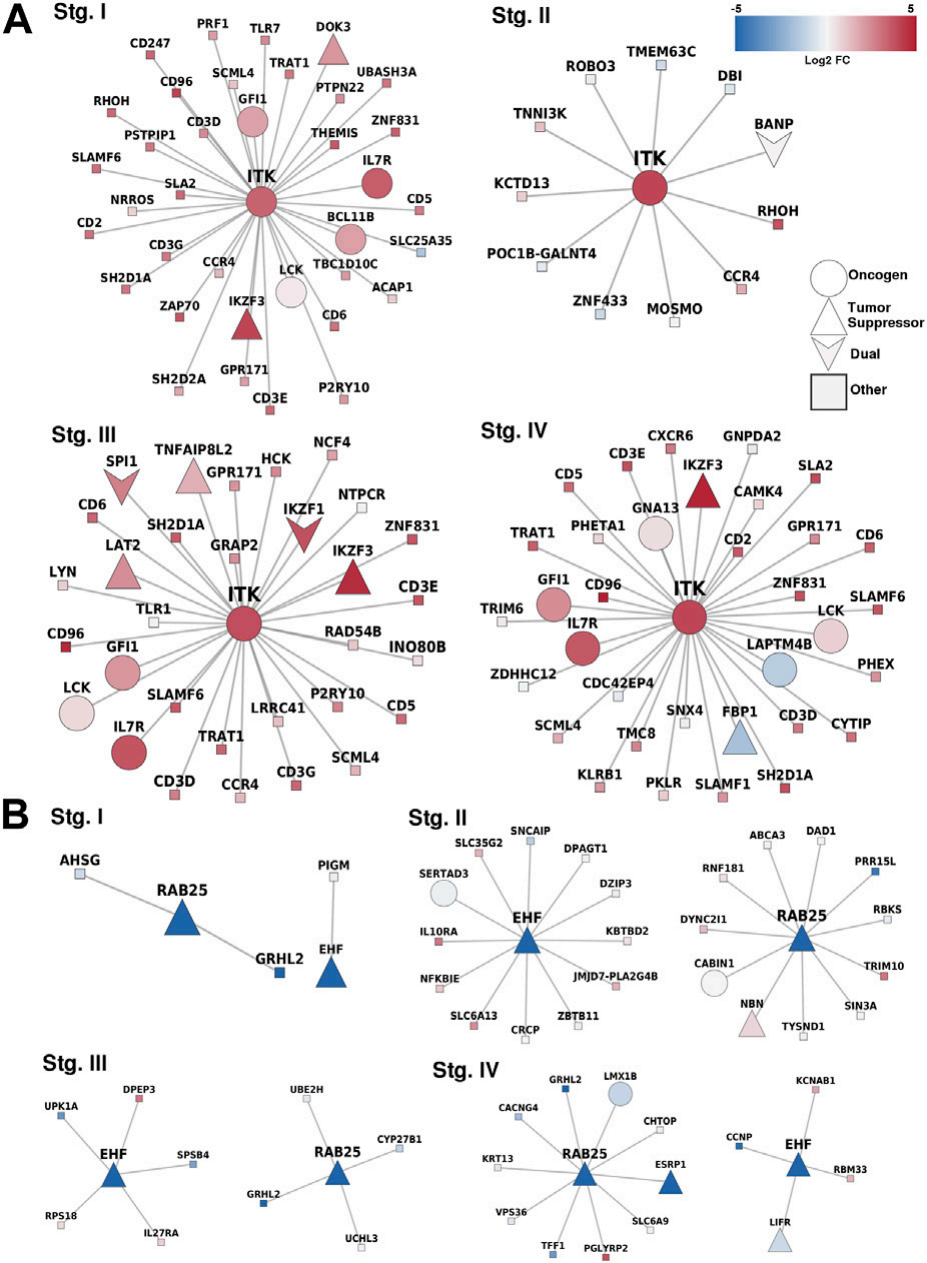
Correlated genes for methylation-related genes. **(A)** Overexpressed-hypomethylated genes. ITK was the only gene found fulfilling our criteria. **(B)** Networks for genes found underexpressed and hypermethylated, in this case, EHF and RAB25. Notice the consistency between the differential expression trend of the MDGs and their first neighbors.

We identified specific methylation-related genes in the four progression stages of ccRC, including IL32, CD68, EHF, and MUC15. These genes were labeled as oncogenes (OGs), tumor suppressor genes (TSGs), or both (if evidence supported both features). Only three genes, namely ITK, RAB25, and EHF, met the criteria of having these properties. To gain a comprehensive understanding of the phenomenon, we constructed a co-expression network involving these genes and their neighboring genes (Figure 5). Additionally, we examined the differential expression patterns of these genes.

With this approach, we identified potential DNA methylation-regulated genes in clear cell renal carcinoma. However, there remains uncertainty regarding the correlation of RAB25 and ITK, with prognosis. Indeed, the correlation between molecular features and cancer prognosis is a cornerstone in cancer research.

### RAB25 and FOXP3 expression are associated with poor prognosis in ccRC

**FIGURE 6:**
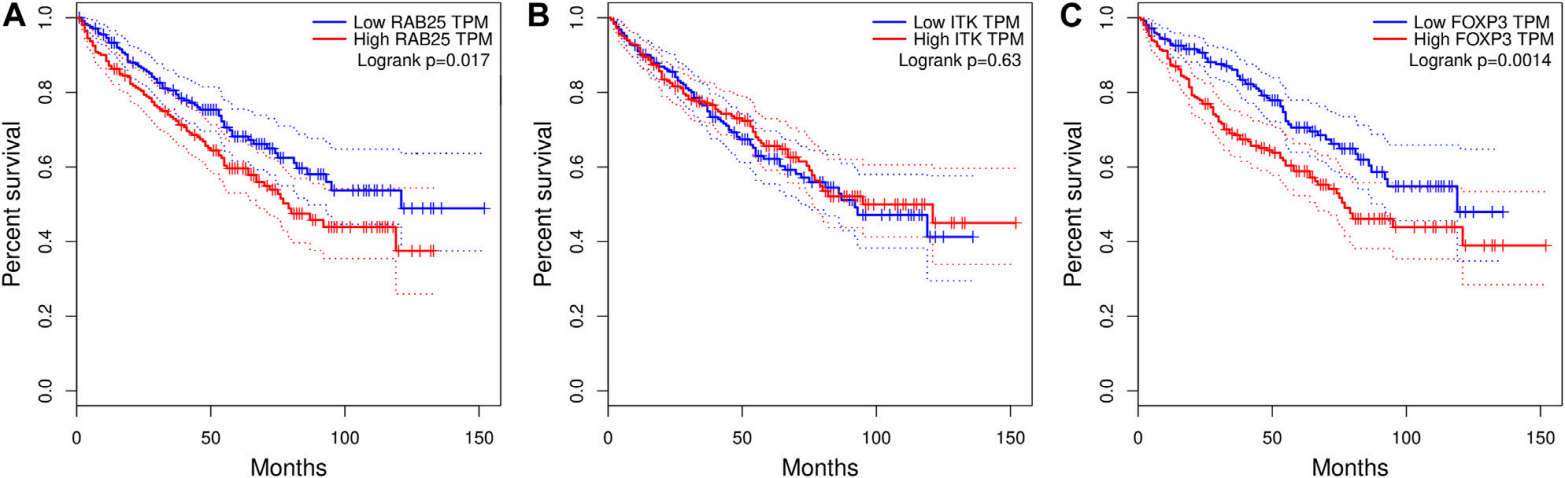
Kaplan–Meier plots correlating methylation-related genes and ccRC survival: **(A)** RAB25 gene. **(B)** ITK. **(C)** FOXP3. The case of FOXP3 is shown here since this gene is a downstream molecule in the ITK signaling pathway but is not modulated by methylation.

In the case of RAB25, we did identify a substantial disparity in prognosis between high- and low-expression levels (Figure 6A). As previously mentioned, RAB25 exhibits dual functionality in carcinogenesis. It acts as an oncogene in some cancer types (Lapierre et al., 2011; Li et al., 2015) while functioning as a tumor suppressor gene in others, such as colorectal cancer, esophageal squamous cell carcinoma, and head and neck squamous cell carcinoma (Goldenring and Nam, 2011; Tong et al., 2012). A Kaplan–Meier curve for RAB25 demonstrates that the high-expression group exhibits a worse prognosis compared to the low-expression group (*p*-value = 0.017).

Regarding ITK gene expression, it is important to note its significant correlation with the prognosis in other cancer types. Previous studies have reported a strong association between ITK expression and poor prognosis in lung adenocarcinoma, breast cancer, hepatocellular carcinoma, and lymphoma (Pan et al., 2021; Liu et al., 2019). However, in these data, we did not observe a significant difference in the prognosis between high- and low-expression ITK levels (Figure 6B). To provide additional insights, we included a Kaplan–Meier plot for FOXP3, a well-known downstream molecule of the ITK signaling pathway (Weeks et al., 2021), which indeed exhibits distinct behavior in relation to prognosis: high expression of FOXP3 is related with the poor prognosis in ccRC (Figure 6C).

## Discussion

With the pipeline performed here, we showed that some genes present a significant association between their expression patterns and the methylation profiles. Some of those genes have been reported to be related with the oncogenic process. Additionally, we showed evidence of the correlation between gene expression and prognosis. In what follows, we will discuss some ideas in light of the aforementioned results.

In terms of the co-expressed genes of ITK, it is worth noting that its first neighbors in the co-expression network have the same expression pattern (overexpressed). ITK encodes an intracellular tyrosine kinase expressed in T cells, which also has a critical role in T-cell growth, signaling, and function. T-cell activation and regulation of the immune system were the two most significantly enriched processes. These results demonstrate the relevance of ITK as this gene maintains its functions independently of its neighbors.

Additionally, ITK codifies for a kinase of T cells. Therefore, the result of overrepresentation analysis where T-cell activation is the most significant process involved in the network of ITK first neighbors reflects an important fact regarding the immune response in ccRC: independent of the progression stage, the hypomethylation of ITK promotes T-cell activation in the cancerous phenotype. The cell of origin in which this hypomethylation occur is a matter of further research.

The case of ITK expression and its correlation with the prognosis is intriguing. ITK is not typically expressed in clear cells within the kidney, suggesting that this gene may originate from immune cells. Moreover, as we stated previously, ccRC is characterized by significant immune infiltration and stromal infiltration, further emphasizing the potential relevance of downstream genes influenced by ITK, such as FOXP3 (Weeks et al., 2021).

In the case of RAB25 and EHF, both genes are well-known tumor suppressors that changed their methylation state. In normal tissues, both were hypomethylated, but their methylation increased in cancer at any stage. As observed, expression patterns in the first neighbors of these genes are similar, either underexpressed or unchanged. This phenomenon may be due to a kind of *anchoring* effect in networks, in which the neighbors follow gene co-expression patterns that affect their individual expression through some regulatory mechanism such as transcription factors, close methylation, or conformational effects (Vipin et al., 2018).

The oncogenic function of Rab25 is likely attributed to its role in regulating vesicle trafficking, which increases integrin recycling to the plasma membrane and stimulates intracellular signaling pathways associated with oncogenic functions (Agarwal et al., 2009). Notably, the loss of Rab25 in human colon cancers has been linked to poorer patient prognosis (Nam et al., 2010).

Furthermore, the reduced expression of RAB25 was shown to correlate with the decreased overall survival and was documented in esophageal squamous cell carcinoma (EScc) cell lines compared to pooled normal tissues (Tong et al., 2012). RAB25 expression in both EScc cell lines and clinical samples was found to be associated with promoter hypermethylation (Gu et al., 2017). The protein encoded by RAB25 is a member of the RAS superfamily of small GTPases (Mitra et al., 2012) and is involved in membrane trafficking and cell survival (Wang et al., 2017). This gene was found to act as a tumor suppressor and also as an oncogene, depending on the context (Mitra et al., 2012). Two variants, one protein-coding and the other non-coding, were identified for this gene (Agarwal et al., 2009).

We showed that epithelium-related processes were enriched, as observed in Supplementary Material S2. This result adds evidence to the often-discussed impacts of extracellular matrix modifications in tumor evolution (Pickup et al., 2014; Frantz et al., 2010; Espinal-Enríquez et al., 2015; Winkler et al., 2020). In this case, RAB25 is somehow losing its functionality due to its underexpression accompanied by its co-expressed components.

It is important to emphasize the RAB25 underexpression in ccRC samples. In terms of prognostic value, high RAB25 expression is associated with an unfavorable outcome, but its expression is regulated by the methylation profile within those samples. One can hypothesize that the methylation of RAB25 may impede its overexpression, thereby influencing prognosis.

As mentioned in Results, we identified methylation-related genes that were found to be overexpressed and hypomethylated in association with the progression stage in ccRC. These genes include IL32, TNFRSF9, LGALS2, CD68, IFI16, and LINE1. Remarkably, these genes exhibited significant overexpression throughout all progression stages while also being significantly hypomethylated with *β*-values below 0.4. Notably, these genes are associated with immune system processes, aligning with the role of ITK in ccRC progression.

For example, IL32 overexpression was identified as a prognostic factor in patients with localized ccRC (Lee et al., 2012). Similarly, IL32 has been suggested to show a positive correlation between its expression and the corresponding methylation state in skin cutaneous melanoma (Kang and Kim, 2021).

Regarding the TNFRSF9 gene, its overexpression has been associated with the progression and prognosis in ccRC (Li et al., 2020b). Moreover, it has been found to be inversely correlated with DNA methylation at various CpG sites in melanoma. Elevated TNFRSF9 mRNA expression and TNFRSF9 hypomethylation were linked to superior overall survival (Fröhlich et al., 2020).

LGALS2 overexpression has been linked to a better prognosis in breast cancer (Chetry et al., 2022). Additionally, PM_2.5_ exposure was positively associated with the methylation of LGALS2-eMS cg07855639 and negatively associated with LGALS2 mRNA expression in monocytes in a diverse population cohort (Chi et al., 2016).

In the case of the CD68 gene, high levels of CD68 are associated with higher tumor grade, larger tumor size, Ki67 positivity, and other malignant features, indicating tumor progression and aggressiveness (Zhang et al., 2022). The methylation profile and its relationship with expression have been associated with prognosis in papillary renal cell carcinoma (Liu et al., 2020). However, its relation with progression in this type of cancer has not been previously reported.

Last, IFI16 promotes cervical cancer progression through the NF-kB pathway (Cai et al., 2020). The expression of this gene has also been correlated with the methylation state in breast cancer cell lines (Khan et al., 2022).

Despite these genes being observed in relation to different types of cancer and their methylation profile showing a correlation with gene expression, a distinct correlation between methylation and gene expression during cancer progression has not been reported.

In this study, we demonstrated that the co-expression networks formed by methylation-related genes consistently differ between progression stages, as shown in Figure 5. Therefore, it can be inferred that the methylation-related genes observed during ccRC stages act differently at each progression stage, and each stage is affected differently by these methylation-related genes.

We argued that changes in methylated genes may be associated with the progression of cancer in at least two general ways: 1) genes that change their methylation/expression status in every stage of cancer or 2) genes whose methylation/expression status remains unaffected in all stages. These epigenetic fingerprints can be studied as biomarkers in a prospective analysis (Vasudev et al., 2012). We suggest a relationship between co-expression and methylated genes during cancer progression. Since methylation can repress gene expression, we can infer gene networks of DMGs for each tumor stage/phenotype. As a result, we can relate some biological functions to events marked by epigenetic modifications.

With this systematic and automated approach, we were able to identify individual CpGs associated with candidate genes as methylation-related genes. This association is based on a test comparing DEGs with differentially methylated CpGs. This further supports the evidence of DNA methylation as one of the main factors affecting the changes between tumor stages and carcinogenesis (Morris and Latif, 2017). Based on the latter, we analyzed how methylation affects the co-expression program as a whole. We found evidence that co-expressed gene clusters may activate antitumor defense mechanisms, specific cellular functions such as T-cell activation, and regulation of the immune system (Waldman et al., 2020). On the other hand, we identified underexpressed and hypermethylated genes that resulted in co-expression. This may turn off cell functions and, thus, alter the morphology and differentiation (Morris and Latif, 2017).

In addition to these key cellular functions affected, we found that a reported tumor suppressor gene (RAB25) was hypermethylated and underexpressed in three out of four stages of cancer, via a deregulated FAK-Raf-MEK1/2-ERK signaling pathway (Gopal Krishnan et al., 2020).

We also showed evidence that ITK’s expression is driven by methylation since its hypomethylation in cancer resulted in overexpression. This epigenetic modification may be driving an antitumor response in four stages, activating immune response functions (Sagiv-Barfi et al., 2015). ITK is not currently reported as an oncogene, but similar to RAB25, we proposed ITK as an epigenetic biomarker. According to the work of Zamora-Fuentes et al. (2022), a progressive increase of several chemokines (Zamora-Fuentes et al., 2020) in ccRC progression was observed. In this case, CXCL13 stands out by taking advantage of immune system cell migration. This molecule triggers intracellular pathways, leading to cell migration in lymphatic nodes and endothelial and epithelial tissues (Kazanietz et al., 2019). The importance of the tumor microenvironment is well known (Hanahan, 2022). The fact that the most enriched processes associated with ITK’s first neighbors is T-cell activation suggests us the relevance that immune infiltration exerts on this carcinoma. In this way, bioinformatic approaches have been developed to quantify cell infiltration in tumors, based on molecular signatures (Yoshihara et al., 2013; Aran et al., 2017; Li et al., 2020a). Interestingly, using TCGA-derived data, renal carcinoma was the tumor with more cell infiltration between 14 tissues (Yoshihara et al., 2013).

## Concluding remarks

ccRC is a complex disease, involving multiple layers of complexity. Therefore, in order to gain a comprehensive picture and better understand its progression, origin, evolution, and associated features must be dissected. In previous works, important differences at the cancer progression stages have been found, highlighting phenomena such as the clear bias to co-expression between genes from the same chromosome (Espinal-Enriquez et al., 2017; de Anda-Jáuregui et al., 2019; García-Cortés et al., 2020; Zamora-Fuentes et al., 2020; Andonegui-Elguera et al., 2021; Garcia-Cortes et al., 2022; Zamora-Fuentes et al., 2022), or the differences in the enrichment processes throughout the progression stages in ccRC (Zamora-Fuentes et al., 2020). However, the connection with epigenetic mechanisms remains a key question in the field (Morris and Latif, 2017).

Although reproducibility is a cornerstone of scientific research, validating our findings with another dataset similar to the one we obtained from TCGA poses significant challenges. First, our study utilized two different high-throughput technologies, namely, Illumina Hi-Seq for RNA-Seq data and Illumina HumanMethylation450 (HM450) arrays, for methylation data. Both technologies were carefully matched for each individual in our dataset. Moreover, the samples were stratified based on the progression stage, necessitating the inclusion of clinical information such as the progression stage and vital status. We ensured that each group contained a substantial number of samples for downstream analyses to yield statistically significant results. To the best of our knowledge, no other currently available dataset possesses all of the aforementioned characteristics. However, taking into account the number of matched samples for each phenotype, the technology used for sequencing and the high standards for sample handling from the data sources allows us to have a robust framework to study the changes in gene expression depending on the methylation profiles at different stages in ccRC. At the same time, the astringent statistics, as well as the reproducibility of the computational pipeline, are encouraging to perform this analysis on other cancer tissues from TCGA.

In conclusion, our research highlighted the important role of methylation-related genes in modulating biological functions and contributing to the progression of various carcinomas, including ccRC. Although there is currently no definitive method for establishing clear relations between genetic and epigenetic factors affecting cancer progression, we developed a data-driven approach to identify methylation-related genes and establish their relationship with gene co-expression and methylation-wide-genome regulation patterns. Our analysis identified several genes, including ITK and TSFRN9, that appear hypomethylated and strongly involved in immune response functions throughout all four stages of ccRC progression, as well as tumor suppressor gene RAB25, which is hypermethylated and potentially avoiding repressed functions in the AKT signaling pathway during ccRC evolution. These findings provide important insights into the underlying epigenetic–genetic mechanisms involved in cancer progression.

## Data Availability

The datasets analysed in this study can be found at the GDC data portal https://portal.gdc.cancer.gov/. Computational pipeline to develop all steps in this work can be found at https://github.com/josemaz/kirc-methyl.git. All figures are deployed with the source code. Gene networks were generated with this code: https://github.com/CSB-IG/ARACNE-multicore.git. Dataset to start pipeline was published in https://zenodo.org/record/7988316#.ZHbSrOzMKrx. The results published here are in whole or part based on data generated by the TCGA Research Network: https://www.cancer.gov/tcga.
