# Supplementary figures and images for: Methylation-related genes involved in renal carcinoma progression
Source: Front Genet. 2023 Aug 25;14:1225158. doi: 10.3389/fgene.2023.1225158 (PMC10486271; doi:10.3389/fgene.2023.1225158)

DNA methylation heatmap

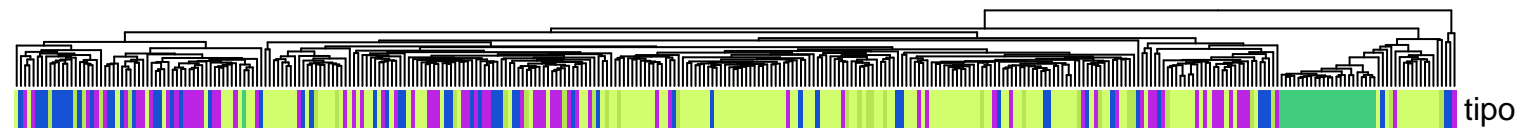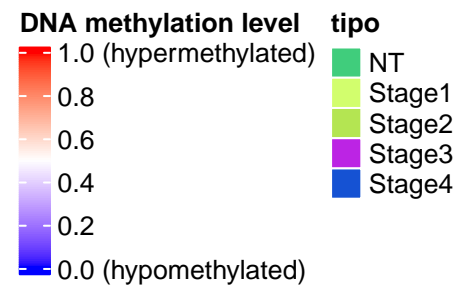

Supplement: Supplementary file 2 [file DataSheet2.zip › heatmap-383862.pdf]

DNA methylation heatmap

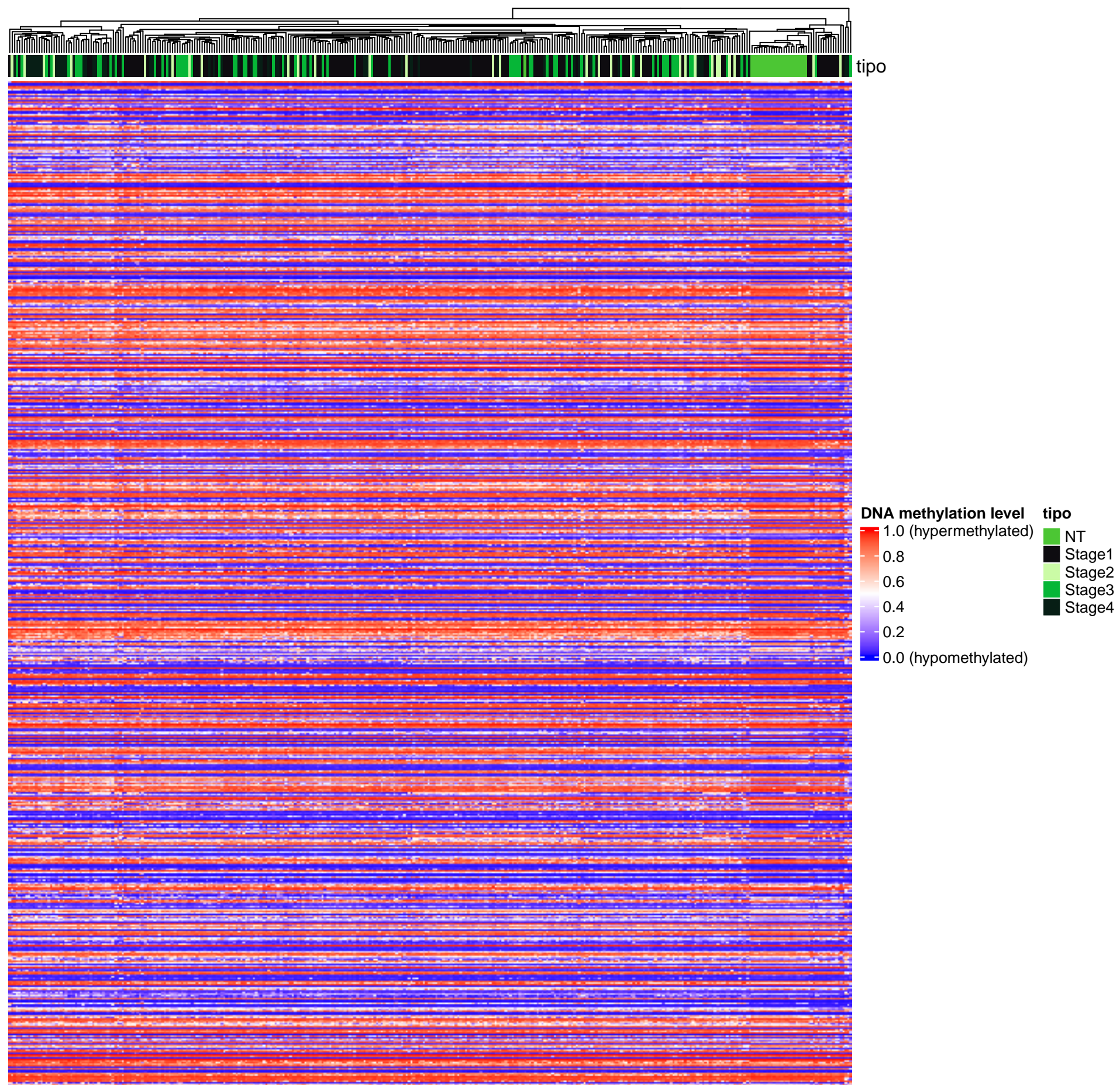

Supplement: Supplementary file 2 [file DataSheet2.zip › heatmap-10000.pdf]

DNA methylation heatmap

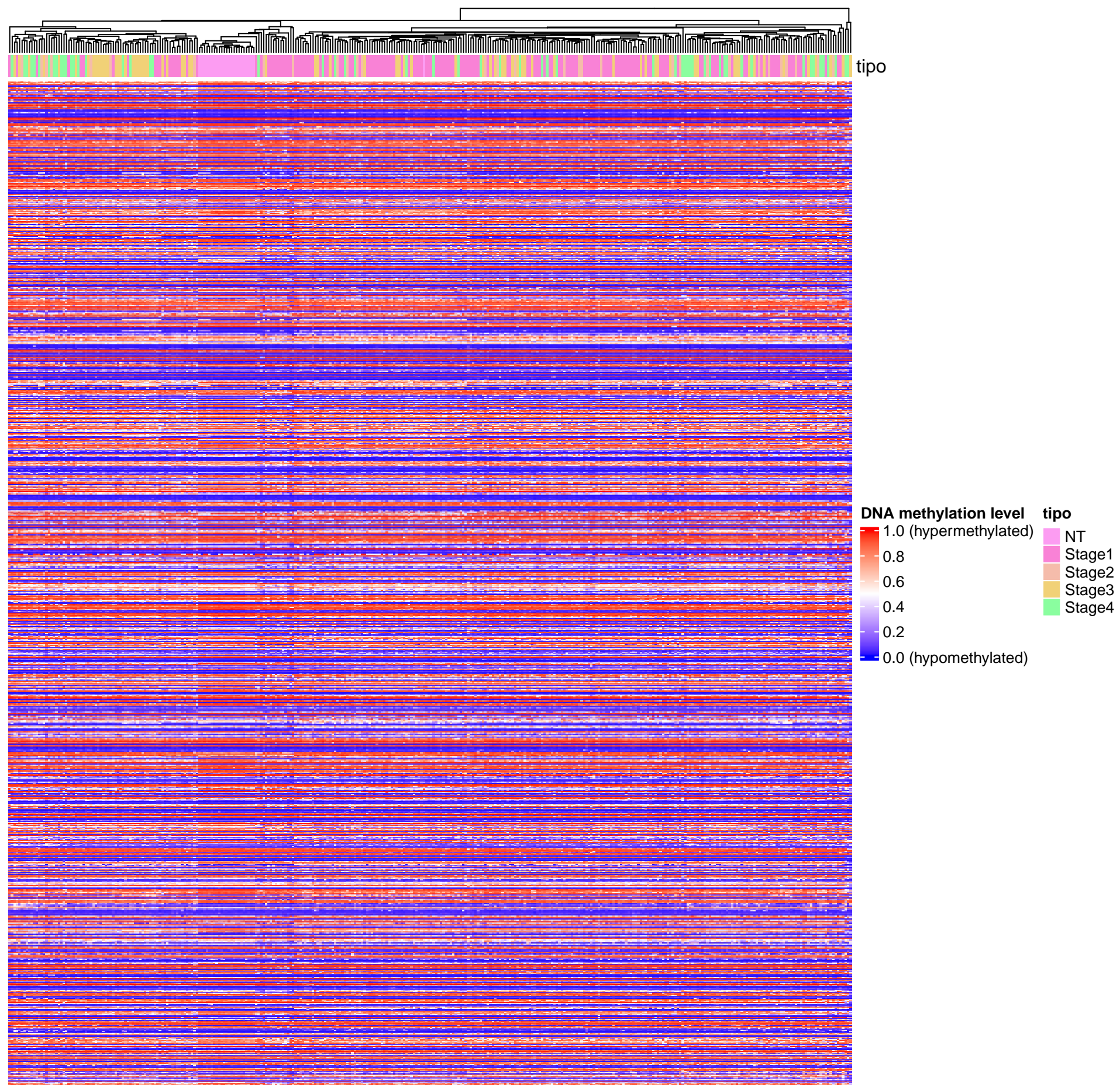

Supplement: Supplementary file 2 [file DataSheet2.zip › heatmap-1000.pdf]

DNA methylation heatmap

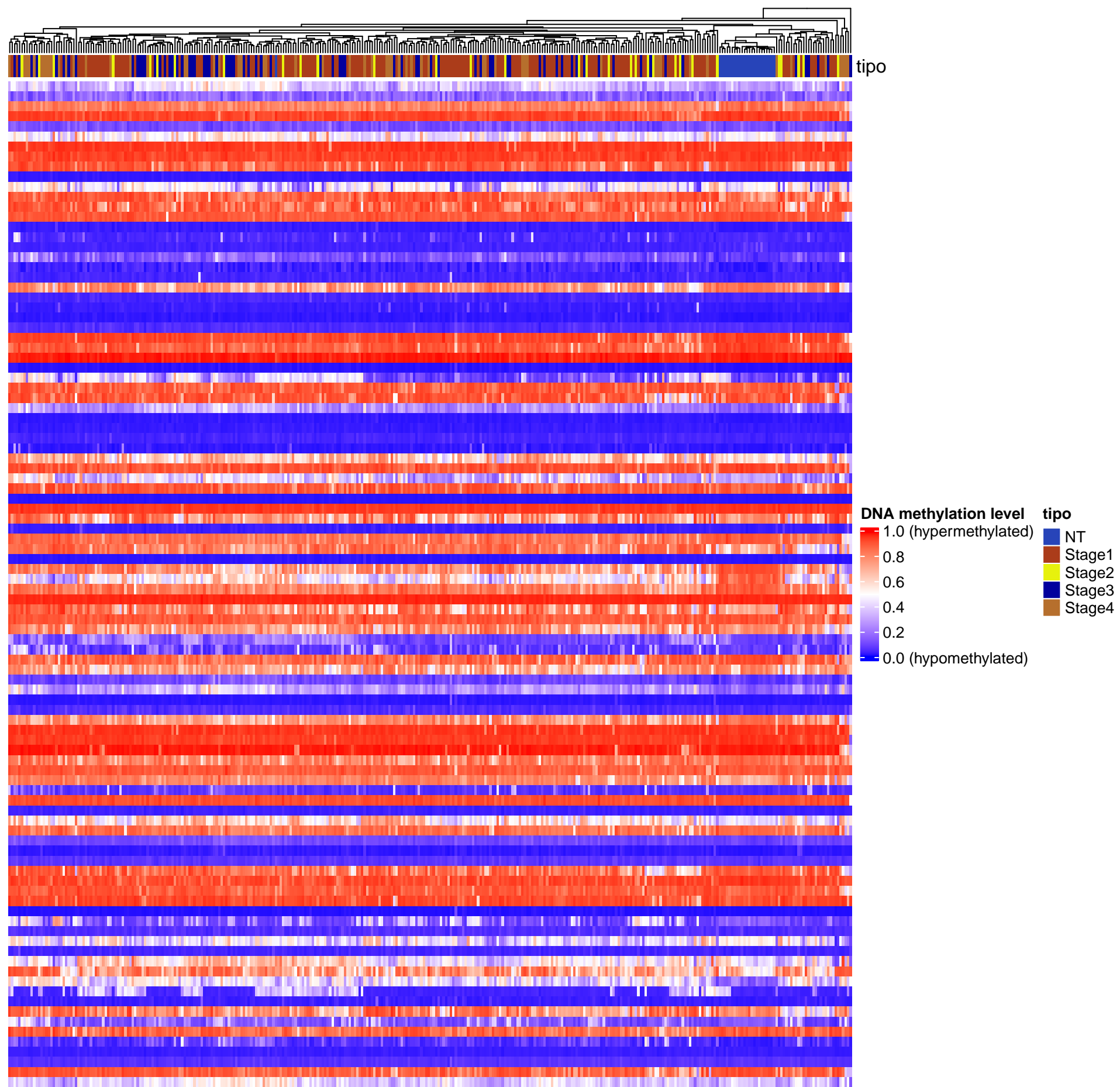

Supplement: Supplementary file 2 [file DataSheet2.zip › heatmap-100.pdf]

DNA methylation heatmap

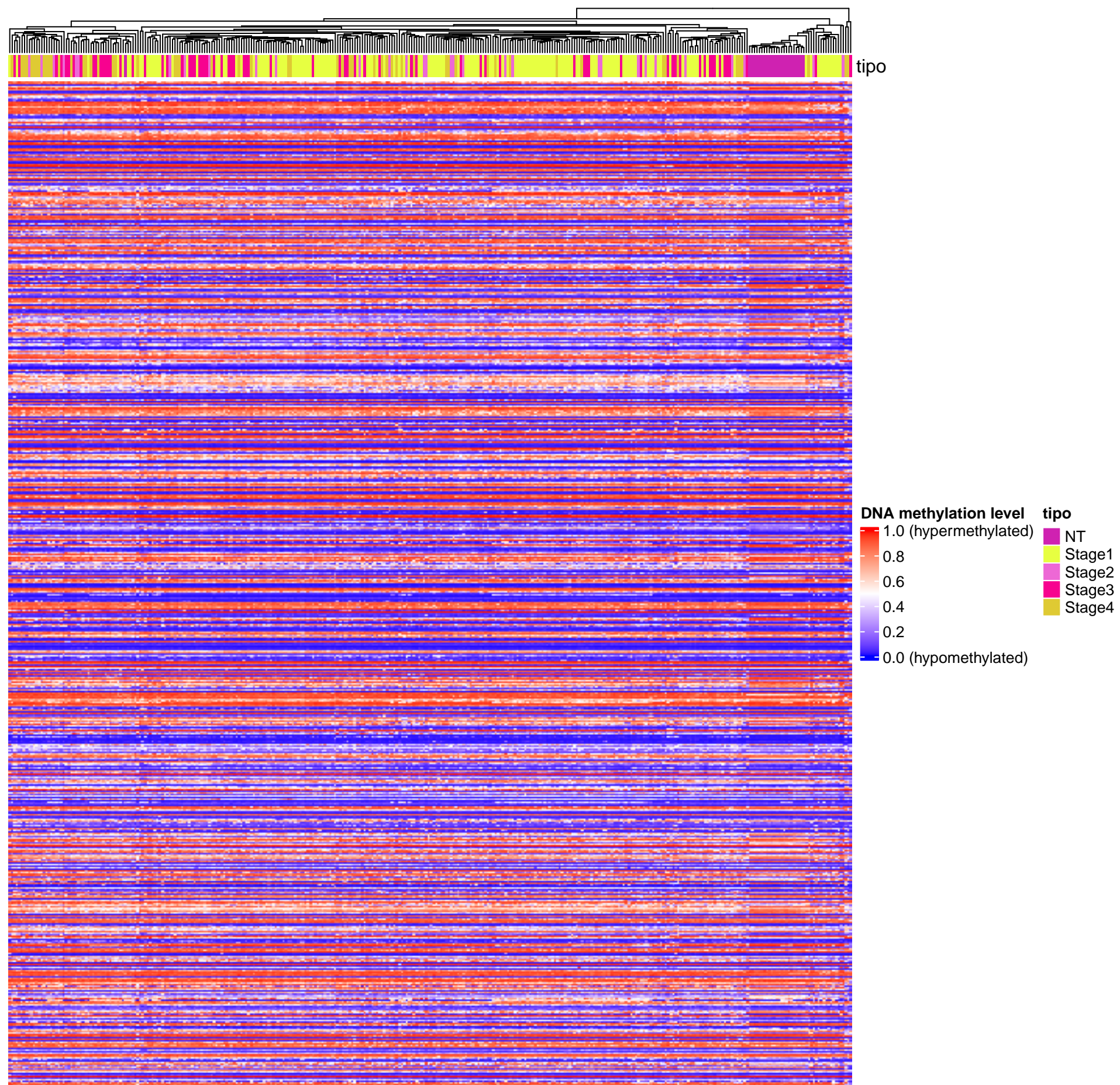

Supplement: Supplementary file 2 [file DataSheet2.zip › heatmap-1e+05.pdf]
